# Transient Receptor Potential Ankyrin 1 Mediates Hypoxic Responses in Mice

**DOI:** 10.3389/fphys.2020.576209

**Published:** 2020-10-22

**Authors:** Sichong Chen, Nobuaki Takahashi, Changping Chen, Jordan L. Pauli, Chiharu Kuroki, Jun Kaminosono, Hideki Kashiwadani, Yuichi Kanmura, Yasuo Mori, Shaowu Ou, Liying Hao, Tomoyuki Kuwaki

**Affiliations:** ^1^Department of Physiology, Graduate School of Medical and Dental Sciences, Kagoshima University, Kagoshima, Japan; ^2^Department of Pharmaceutical Toxicology, School of Pharmacy, China Medical University, Shenyang, China; ^3^Department of Synthetic Chemistry and Biological Chemistry, Graduate School of Engineering, Kyoto University, Kyoto, Japan; ^4^The Hakubi Center for Advanced Research, Kyoto University, Kyoto, Japan; ^5^Department of Neurosurgery, First Affiliated Hospital of China Medical University, Shenyang, China; ^6^Department of Anesthesiology and Critical Care Medicine, Graduate School of Medical and Dental Sciences, Kagoshima University, Kagoshima, Japan

**Keywords:** transient receptor potential ankyrin-1, avoidance behavior, hypoxic arousal, respiratory chemoreflex, trigeminal afferent nerve

## Abstract

Transient receptor potential ankyrin 1 (TRPA1) is a non-selective cation channel that is broadly expressed in sensory pathways, such as the trigeminal and vagus nerves. It is capable of detecting various irritants in inspired gasses and is activated during hypoxia. In this study, the role of TRPA1 in hypoxia-induced behavioral, respiratory, and cardiovascular responses was examined through four lines of experiments using TRPA1 knockout (KO) mice and wild type (WT) littermates. First, KO mice showed significantly attenuated avoidance behavior in response to a low (15%) oxygen environment. Second, the wake-up response to a hypoxic ramp (from 21 to 10% O_2_ in 40 s) was measured using EEG electrodes. WT mice woke up within 30 s when oxygen was at 13–14%, but KO mice did not wake up until oxygen levels reached 10%. Histological analysis confirmed that mild (13% O_2_) hypoxia resulted in an attenuation of trigeminal neuronal activation in KO mice. Third, the ventilatory response to hypoxia was measured with whole body plethysmography. KO mice showed attenuated responses to mild hypoxia (15% O_2_) but not severe hypoxia (10% O_2_). Similar responses were observed in WT mice treated with the TRPA1 blocker, AP-18. These data clearly show that TRPA1 is necessary for multiple mild hypoxia (13–15% O_2_)-induced physiological responses. We propose that TRPA1 channels in the sensory pathways innervating the airway can detect hypoxic environments and prevent systemic and/or cellular hypoxia from occurring.

## Introduction

A supply of oxygen is essential for aerobic animals, and maintaining it is a matter of life and death. There are several levels of regulatory systems that monitor oxygen supply. For example, the carotid body is a small organ that responds to decreases in partial pressure of oxygen in arterial blood, or hypoxemia, by increasing respiration (Prabhakar and Semenza, 2015), and hypoxia inducible factors (HIFs) are transcription factors that respond to decreases in available oxygen in the cellular environment (Choudhry and Harris, 2018). These are examples of feedback control systems, which trigger responses after a change has been detected in their monitored target (in this case, oxygen partial pressure in arterial blood and oxygen concentration in tissue fluid around the cells). A feedforward control system, however, detects an environmental change and triggers responses before an actual effect is able to take place in the monitored target (Mateika and Duffln, 1995). A proper oxygen supply is critically important so it stands to reason that a feedforward control system should participate in its regulation.

Transient receptor potential ankyrin 1 (TRPA1) is a non-selective cation channel that is likely to be involved in regulation of a feedforward system that monitors oxygen supply. TRPA1 is expressed in sensory nerves including those that innervate the respiratory tract, such as the trigeminal and vagus nerves (Takahashi et al., 2011; Yonemitsu et al., 2013). TRPA1 detects irritants such as pungent chemicals found in onion, garlic, wasabi, tobacco smoke, and predator odor in inspired gasses, and induces escape behavior or respiratory arrest (Yonemitsu et al., 2013; Akahoshi et al., 2016; Inui et al., 2016; Wang et al., 2018). We have previously reported that nasal TRPA1 is important for detection of airborne irritant by showing lack of escaping behavior from formalin vapor by nasal but not subcutaneous application AP18, one of the blockers of TRPA1 (Yonemitsu et al., 2013). Allyl isothiocyanate, a component of mustard and wasabi, activated trigeminal ganglion neurons in wild type (WT) mice but not in TRPA1-KO mice (Inui et al., 2016). These reports indicated importance of trigeminal TRPA1 for irritant detection.

TRPA1 also detects oxygen concentration, and its sensitivity is the highest in the TRP family molecules *in vitro* (Takahashi et al., 2011). TRPA1 is activated by hyperoxia through direct modification of cysteine residues by oxygen (Takahashi et al., 2011). Under normoxia, TRPA1 is inhibited through prolyl hydroxylation by prolyl hydroxylase domain (PHD) enzymes (Takahashi et al., 2011) that are related to the hypoxia-inducible factor HIF-1. TRPA1 is activated by hypoxia through inactivation of PHD and de-hydroxylation of prolyl residues (Takahashi et al., 2011). Of note, TRPA1 is most inactive at normal atmospheric oxygen partial pressure (~150 mmHg) and almost maximally activated at oxygen partial pressure less than 80 mmHg and more than 300 mmHg (Takahashi et al., 2011). These molecular characteristics support our hypothesis but *in vivo* evidence for participation of TRPA1 in sensing oxygen in the inhaled gas is still sparse (Pokorski et al., 2014). In order to obtain more comprehensive evidence for participation of TRPA1 in oxygen sensing and possible physiological role of TRPA1 *in vivo*, we examined hypoxia-induced behavioral, respiratory, and anatomical changes in TRPA1-KO mice and in wild type (WT) mice.

## Materials and Methods

### Ethics Approval

All experiments were conducted at Kagoshima University in accordance with the guiding principles for the care and use of animals in the field of physiological sciences published by the Physiological Society of Japan (2015) and were approved by the Experimental Animal Research Committee of Kagoshima University (MD13007 and MD17105). All efforts were made to minimize the number, and reduce the pain of animals.

### Animals

TRPA1 knockout (KO) mice were purchased from Jackson Laboratory and genotyped as previously described (Kwan et al., 2006). Mice were maintained as heterozygotes in our facility and crossed to obtain null mutants and wild type (WT) littermates (male, 12–24 weeks old). TRPA1 KO mice were backcrossed with C57BL/6 mice (CLEA, Japan) for more than 10 generations and were thought of as congenic to C57BL/6. In this study, 48 KO mice and 73 WT mice were used.

### Immunohistochemistry

#### Distribution of TRPA1 in the Nasal Cavity

**Figure 1:**
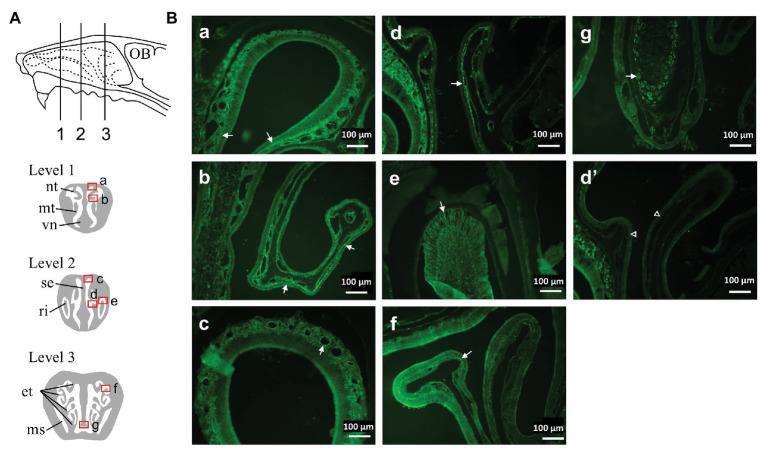
Distribution of Transient receptor potential ankyrin 1 (TRPA1) immunoreactivity in the nasal cavity of mouse. **(A)** Schematic representation of the lateral view of the mouse nasal area showing three levels of the coronal sections examined. **(B)** Photomicrographs taken from the areas indicated by squares **(a–g)** in **(A)** of a representative mouse. Arrows indicate TRPA1-positive structures (see text). **(d’)** was taken from a similar region to **(d)** and treated with a mixture of anti-TRPA1 antibody and an excess amount of antigen peptide. Similar results were obtained in three animals. et, ethmoturbinate; ms, maxillary sinus; mt, maxilloturbinate; nt, nasoturbinate; OB, olfactory bulb; ri, root of incisor tooth; se, septum; vn, vomeronasal organ.

Wild type mice were deeply anesthetized with urethane (2.0 g/kg) and transcardially perfused with 0.01 mol/L phosphate-buffered saline (PBS) followed by a fixative solution containing 4% paraformaldehyde in PBS. The head was dissected and immersed in the same fixative solution for 24 h at 4°C. After removal of the skin and mandibula, the head block was immersed in 0.5 M EDTA in PBS for decalcification for 1 week. After cryoprotection with 30% sucrose in PBS, a coronal block (thickness~5 mm) was made and immersed in O.C.T. compound (Sakura Finetek, Tokyo, Japan). Before freezing, a negative pressure was applied to the specimen for better penetration of the compound into the narrow and complex structure of the nasal meatus (Dunston et al., 2013). Serial coronal sections of 16 μm thicknesses were cut using a freezing microtome and mounted onto a slide glass (Platinum pro, Matsunami, Osaka, Japan). The sections were immunohistochemically stained for TRPA1 (1/1,000, raised in rabbit, ACC-037, Alomone labs) using a slide rack immunostaining system (Shandon Sequenza, Thermo Fisher Scientific) and visualized with a CF488-conjugated donkey anti-rabbit IgG antibody (1/500, #20015, Biotium). Specificity of the anti-TRPA1 antibody was examined by pre-mixing with an excess amount of antigen peptide (weight ratio 1:1, which corresponds molar ratio of ~1:100, Alomone) for 60 min. The sections were examined with a fluorescence microscope (BZ-8000, Keyence, Osaka, Japan) according to the position in the nasal cavity (Figure 1A).

#### Activation of Trigeminal Ganglion Neurons

To examine possible activation of the trigeminal nerve by hypoxia, we used immunohistochemical detection of the phosphorylated form of extracellular signal-regulated kinase (p-ERK) in trigeminal ganglion neurons. P-ERK is a cellular activation marker that has a more rapid and narrow time window than that of other activation markers including c-Fos (Antoine et al., 2014). We have previously shown that allyl isothiocyanate, one of the activators of TRPA1, activates trigeminal neurons in WT but not in TRPA1-KO mice (Inui et al., 2016). For this purpose, mice exposed to hypoxia (13% or 10% O_2_) for 3 min were immediately anesthetized with urethane (1.8 g/kg) and transcardially perfused as described in the above section. The trigeminal ganglia were dissected and post-fixed for 24 h at 4°C. After cryoprotection with 30% sucrose in PBS, 16 μm thick serial longitudinal frozen sections were cut, and every fourth section (10 slices/animal) was immunohistochemically stained for p-ERK (1/400, raised in rabbit, #4370S, Cell Signaling Technology) and a neuronal marker, NeuN (1/400, raised in guinea pig, #266004, Synaptic Systems). P-ERK was visualized with a biotinylated donkey anti-rabbit IgG antibody (1/250, #711-065-152, Jackson Immunoresearch) and streptavidin-conjugated Alexa Fluor 488 (1/200, S11223, Invitrogen). NeuN was visualized with a CF568-conjugated anti guinea pig IgG antibody (1/200, raised in donkey, #20377, Biotium). The number of labeled cells was counted in a manner blinded to the treatment.

#### Behavioral Tests

All behavioral experiments were performed in a quiet, air-conditioned (25 ± 1°C) room from 10:00 to 16:00, during the mouse’s inactive cycle. The mouse was acclimatized to the measuring chamber at least three times prior to the experimental day.

#### Hypoxia Avoidance Test

**Figure 2:**
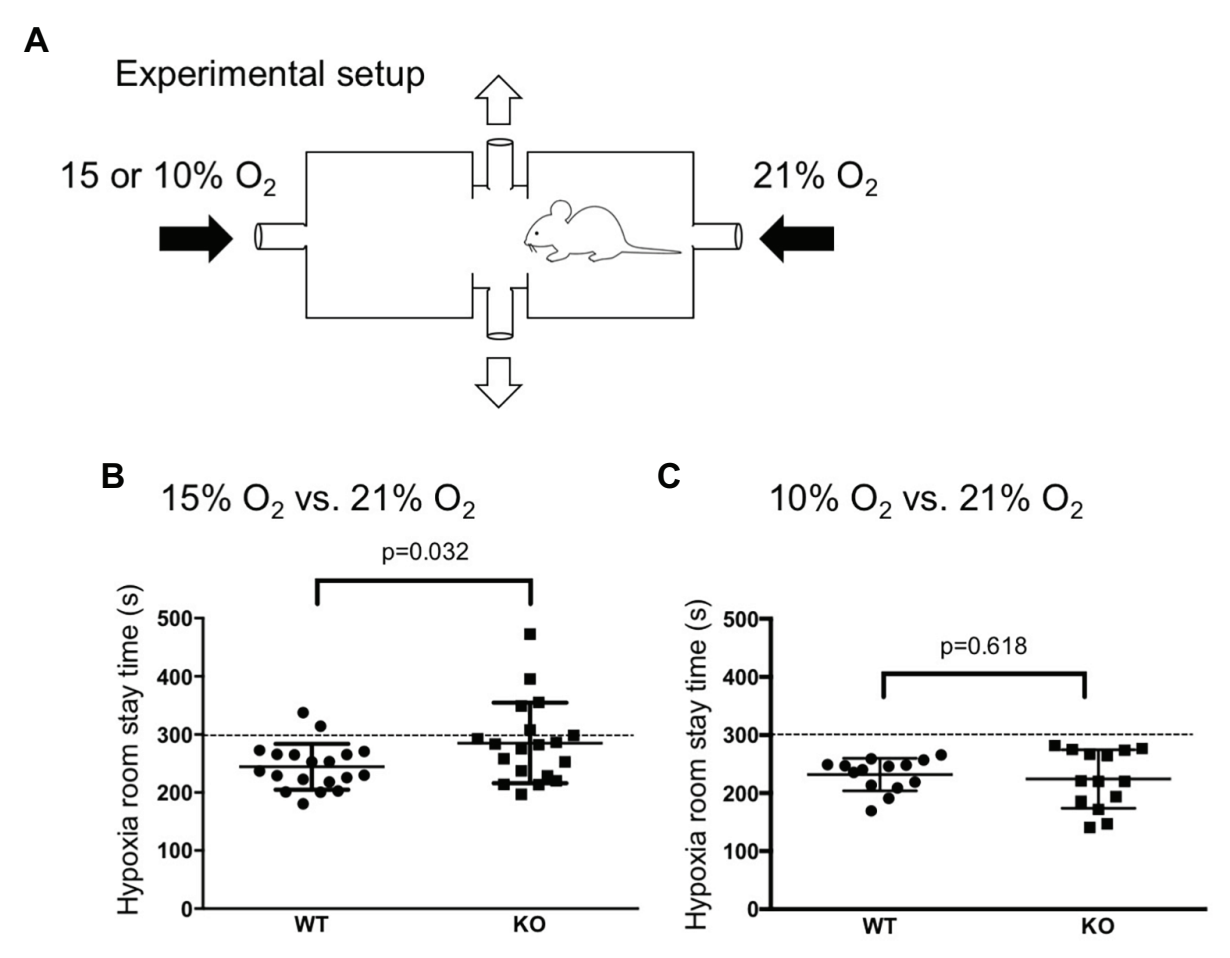
Hypoxia avoidance test. **(A)** Illustration of an apparatus to create an oxygen concentration gradient in which a mouse can move back and forth (see method section for detail). **(B,C)** WT mice (*n* = 19) avoided both mild (15% O_2_, **B**) and severe (10% O_2_, **C**) hypoxia whereas knockout (KO) mice (*n* = 19) avoided only severe hypoxia. The dotted lines at 300 s indicates chance level during the observation period of 10 min. Horizontal lines indicate mean and SEM. The difference between the two genotypes was assessed using the Student’s *t*-test, and value of *p* is shown.

We used a home-made oxygen gradient chamber (Figure 2A). Two plastic boxes (1.2 L) with a roof door were connected *via* a tube that allowed the animal to freely move back and forth. Room air (21% O_2_) and hypoxic gas (15 or 10% O_2_) were introduced (0.8 L/min) to the opposite chambers from the side, and the gas mixture was removed from the chamber through a hole in the connecting tube in the center. In a preliminary experiment without animals in the chamber, we confirmed that the oxygen concentration near the inlet of the room air, the inlet of the 15% O_2_, and the center of the connecting tube was 21, 15, and 17%, respectively, 10 min after the chamber roof was closed. Oxygen concentration was monitored with an O_2_ sensor (JKO-25LJII, JIKCO, Japan). After a period of time allowing for an equilibrium in the oxygen gradient in the chamber to be established (>15 min), the roof of the room air-inlet side was briefly opened, and a mouse was placed in the chamber. Animal behavior was observed for 10 min.

#### Simultaneous Measurement of EEG and Ventilation by Whole Body Plethysmography

**Figure 3:**
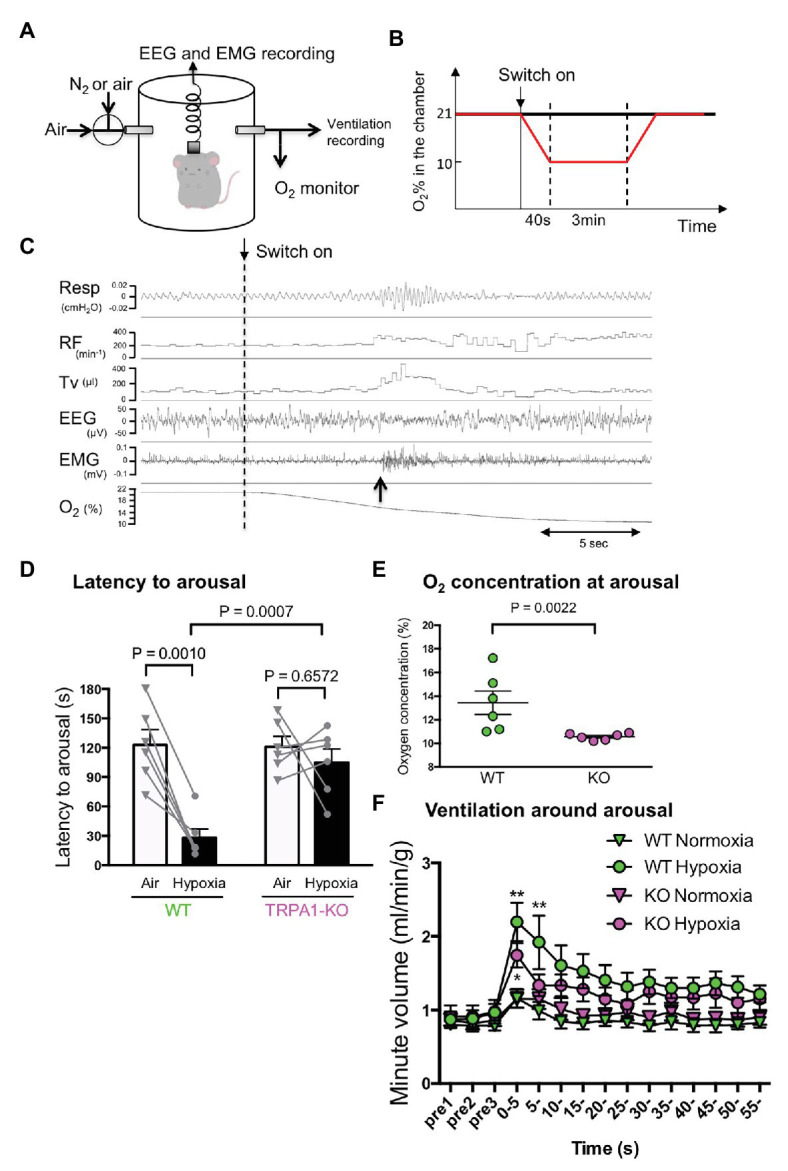
Hypoxia-induced wakeup test. **(A)** Simultaneous measurement of EEG, EMG, and ventilation by whole body plethysmography. Continuous measurement was obtained using flow-through type body plethysmography. **(B)** Experimental schedule used to apply hypoxia to mice. **(C)** Typical recording from a WT mouse showing ventilatory parameters (RF, respiratory frequency; TV, tidal volume), electrophysiological measures, and O_2_ concentration in the chamber. The dashed vertical line shows the timing when the inflow was changed from room air to hypoxic (10% O_2_) gas. The upward arrow shows the timing when the mouse woke up as judged by EEG and EMG. Note that the increase in ventilation was not associated with the start of hypoxia but with the timing of arousal. **(D)** Latency to arousal from the time of inflow change. The bar indicates mean and SEM, *n* = 6 for both WT and KO mice. Two-way ANOVA revealed a significant difference between genotypes (*F*1,10 = 9.851, *p* = 0.0105) and between gas conditions (*F*1, 10 = 17.24, *p* = 0.0020). Values of *p* from Sidak’s multiple comparison test are shown. **(E)** O_2_ concentration in the chamber at arousal. A nonparametric Mann-Whitney U-test was used to compare WT and KO mice because data in KO mice were not normally distributed. **(F)** Minute ventilation was calculated by RF × TV, normalized with body weight and averaged every 5 s with arousal timing set to be time zero. Two-way ANOVA revealed there was a significant difference among times (*F*14, 280 = 16.20, *p* < 0.0001) but not among genotypes × gas conditions (*F*3, 20 = 2.786, *p* = 0.0673).

Following previously published methods (Nakamura et al., 2003; Deng et al., 2007; Terada et al., 2007; Iwakawa et al., 2019), electroencephalography (EEG) and electromyography (EMG) were recorded during ventilation measurement by whole body plethysmography in freely moving mice (see also Figure 3A). EEG and nuchal EMG electrodes were implanted at least 2 weeks prior to the experiment. An animal’s vigilance state was manually judged by EEG and EMG recordings. Plethysmographic signals were recorded as changes of the pressure in the recording chamber and transformed into tidal volume according to a previously reported method (Onodera et al., 1997; Nakamura et al., 2003). Chamber was continuously flushed with room air or mixed gas of room air and N_2_. O_2_ concentration was continuously monitored with an O_2_ sensor at the outlet of the chamber.

#### Hypoxia-Induced Wakeup Test

Mice were placed in a plethysmography chamber (700 ml) that was continuously flushed with room air (500 ml/min). When animals fell asleep (slow wave sleep) for more than 1 min, the inlet gas was switched to a hypoxic gas mixture (10% O_2_ balance with N_2_) at a rate of 500 ml/min (Figure 3B). When the O_2_ concentration in the chamber reached 10%, the condition was continued for 3 min before being switched back to the room air. For the control, the process remained exactly the same with the exception that room air was introduced into the chamber and not hypoxic gas. Each animal was given four trials of hypoxia and four trials of control with an interval of >10 min in a random order. The average value of the four trials was treated as the value for each animal.

#### Measurement of Hypoxic Chemoreflex Using Whole Body Plethysmography

Whole body plethysmographic chamber was continuously flushed with a gas mixture (10–100% O_2_) at a rate of 500 ml/min, and change of pressure in the chamber was continuously monitored in a similar manner to those in hypoxia-induced arousal test described above (Figure 3A) except for EEG/EMG recording. After a baseline measurement for 5 min, 100, 15, and 10% O_2_ was introduced into the chamber in this order. Finally, hypercapnic chemoreflex was measured in 5% CO_2_–21% O_2_-residual N_2_. Each test gas continued for 3 min, and the intervals between each exposure was at least 20 min until when respiratory parameters (frequency and tidal volume) returned to the value within ±10% of the baseline value. In the test period of 3 min, values during the last 20-s were averaged and used for further analysis.

#### Aerosol Administration of a TRPA1 Blocker, AP-18, During Body Plethysmography

AP-18 (ab144587, Abcam, Cambridge, England) was dissolved in 20% ethanol in saline. AP-18 was aerosolized with an ultrasonic nebulizer (NE-U17, Omron, Kyoto, Japan), mixed with air, and subsequently distributed into the whole-body plethysmography chamber. At the time of measurement, the supply of air and monitoring of O_2_ were paused for 3 min by closing the inlet and outlet stopcocks of the chamber to avoid fluctuation of the basal gas flow from vibration of the nebulizer because accurate recording of respiration by the flow-through type whole body plethysmography depends on constant air flow (Onodera et al., 1997).

#### Isolation of Carotid Body and RNA Measurement by RT-PCR

Carotid body was excised under dissecting microscope from decapitated mice. Dorsal root ganglia (DRG) were also sampled as the control. Total RNA was extracted using ISOGENE (Nippon Gene, Tokyo, Japan) following the manufacturer’s instructions. The concentration and purity of RNA were determined spectrophotometrically. Two hundred nanograms of total RNA were reverse-transcribed into first-strand cDNA by use of the RNA LA PCR kit at the final volume of 20 μl. PCR was conducted with a GeneAmp PCR system 9700 (Applied Biosystems) using LA Taq polymerase with GC buffer (Takara Bio, Kusatsu, Japan) for 32 cycles under the following conditions: initial denaturation was 3 min at 95°C, then 30 s at 95°C, following by a 30-s annealing step at 55°C and 30-s elongation at 72°C, and a final elongation of 7 min at 72°C. We used primer sets for TRPA1 (forward: ACAAGAAGTACCAAACATTGACACA and reverse: TTAACTGCGTTTAAGACAAAATTC), tyrosine hydroxylase (TH; forward: GGACATTGGACTTGCATCTCTGGG and reverse: GCTTGGGTCAGGGTGTGCAG), and beta-action (forward: GATGACGATATCGCTGCGCTG and reverse: GTACGACCAGAGGCATACAGG). Tyrosine hydroxylase was used as a marker of glomus cells in the carotid body (Mills et al., 1978), and beta-actin was used as an internal standard.

#### Statistical Analyses

All data are expressed as means ± SEM. Statistical analyses were carried out using Prism Software v.6 (GraphPad). We evaluated statistical significance with the Student’s *t*-test or nonparametric Mann-Whitney’s U test (when the data distribution was different between two groups) for comparisons between two mean values. We carried out multiple comparisons among more than three groups with ANOVA followed by Tukey’s or Sidak’s *post hoc* tests. Respiratory parameters were also assessed by ANOVA with repeated measures design. A value of *p* < 0.05 was considered significant.

## Results

### Expression of TRPA1 in the Nasal Cavity

Although many studies have reported on the existence of TRPA1 in the trigeminal ganglion neurons (Kim et al., 2010; Takahashi et al., 2011; Yonemitsu et al., 2013; Inui et al., 2016), distribution in the nasal cavity has not been fully explored (Nakashimo et al., 2010). Therefore, we set out to examine the distribution of TRPA1 in the nasal cavity. TRPA1-like immunoreactivity was found in several structures in the various rostro-caudal levels of the nasal cavity (Figure 1). These include nerve fiber-like structures running under epithelial cell layer (Figures 1Ba,Bb,Bd,Bf), perivasculature (Figure 1Bc), root of the incisor (Figure 1Be), and cells in the septum (Figure 1Bg). Of these, the nerve fiber-like structures were strongly positive in the rostral levels (Figures 1Ba,Bb,Bd) under the respiratory epithelium and relatively sparse in the caudal level (Figure 1Bf), where olfactory epithelium is dominant (Harkema et al., 2006). This distribution was similar to that of trigeminal nerve fibers (Lee et al., 1995). Specificity of the antibody was confirmed by elimination of the positive signal after preincubation of the antibody with an excess amount of antigen peptide (Figure 1Bd’).

### TRPA1-KO Mice Did Not Avoid Mild Hypoxia

We examined whether the mice are able to discriminate between differences in oxygen concentration and avoid a hypoxic atmosphere by using a home-made apparatus (Figure 2A). When there was no oxygen concentration gradient during the acclimatization period, both WT mice and TRPA1-KO mice showed no bias to either chamber and spent equal time in both chambers (data not shown). When an oxygen concentration gradient did exist, WT mice, but not TPRA1-KO mice, avoided the 15% O_2_ room (Figure 2B). When the oxygen concentration gradient was steeper, both WT mice and TPRA1-KO mice avoided the 10% O_2_ room (Figure 2C). This result indicated that the mice can discriminate between the differences in oxygen concentration and avoid hypoxic environments. TRPA1 seems to contribute to detecting mild hypoxia but another mechanism must be involved in the detection of severe hypoxia.

### Hypoxia-Induced Arousal Was Delayed in TRPA1-KO

Hypoxic environments can induce arousal in animals, allowing the body to respond in order to avoid a potentially life-threatening situation (Phillipson et al., 1978). The possible contribution of TRPA1 in hypoxia-induced arousal was examined using a previously established method of simultaneous measurement of EEG, EMG, and ventilation in freely behaving mice (Figure 3A). When mice fell asleep for over 1 min, either hypoxic gas or normal room air was introduced into the recording chamber (Figure 3B), and the wake up time was judged from EEG and EMG recordings (upward arrow in Figure 3C). When hypoxic gas was introduced into the chamber, the latency to wake up in WT mice was significantly shortened (28.0 ± 9.0 s, *n* = 6) from the latency in normoxia (122.7 ± 15.7 s, *p* = 0.0010, Sidak’s multiple comparison test; Figure 3D). Whereas in TRPA1-KO mice, latency to wake up did not change under hypoxia (from 120.8 ± 10.9 s to 104.7 ± 13.9 s, *p* = 0.6572). When a hypoxic challenge was given to the animals, the O_2_ concentration in the chamber reached 10% in 40 s in our experimental setting. Therefore, a latency of >40 s means that the animal did not wake up until O_2_ concentration reached 10%. The O_2_ concentration at arousal in TRPA1-KO mice (10.6 ± 0.1%) was significantly lower than in WT mice (13.4 ± 1.0%, *p* = 0.0022, Mann-Whitney U test; Figure 3E). Hypoxia-induced arousal was associated with a brief increase in ventilation in both WT and KO mice (Figure 3F). Within the first 5 s after arousal, minute ventilation in KO mice (1.744 ± 0.163 ml/min/g, *n* = 6) seemed to be lower than in WT mice (2.196 ± 0.263 ml/min/g, *n* = 6) but the difference did not reach statistical significance (*p* = 0.3623, Sidak’s multiple comparison test). Thus, TRPA1 seems to contribute to mild hypoxia-induced arousal but has less of an effect, if any, on sever hypoxia-associated respiratory augmentation.

### Mild Hypoxia Failed to Activate Trigeminal Ganglion Neurons in TRPA1-KO

**Figure 4:**
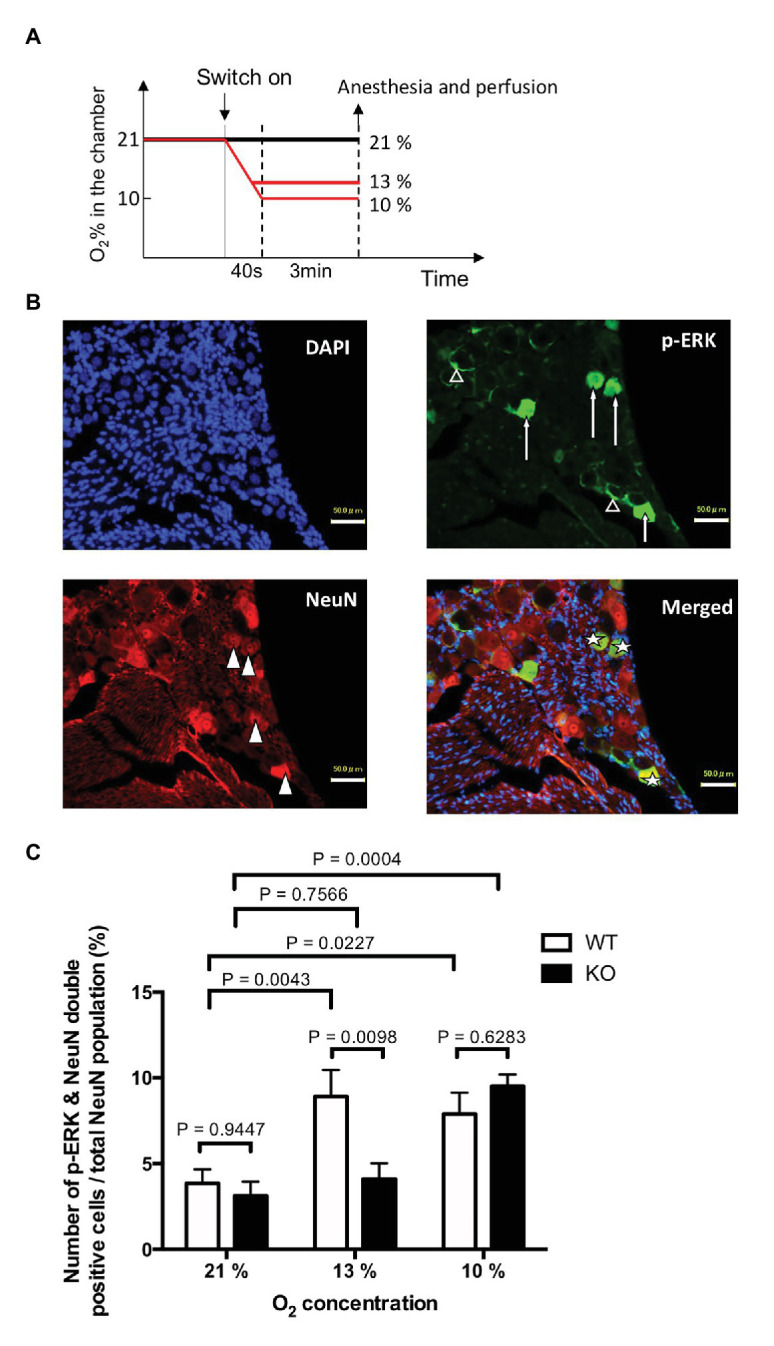
Hypoxia induced activation of trigeminal ganglion neurons. **(A)** Experimental design. TRPA1-KO mice and WT mice were exposed to mild (13% O_2_) or severe (10% O_2_) hypoxia for 3 min, quickly euthanized, and then the trigeminal ganglion was sampled. Room air (21% O_2_) was used as the control. **(B)** Typical example from WT mice that experienced 13% hypoxia. Note that p-ERK was positive in both neurons (arrows) and satellite cells (open triangles). **(C)** Ratio of p-ERK and NeuN double positive cells out of the total NeuN positive population. Each column represents mean and SEM in five animals. Two-way ANOVA revealed that there was a significant difference among O_2_ concentrations (*F*2, 24 = 12.67, *p* = 0.0002) and interaction between O_2_ concentration and genotype (*F*2, 24 = 4.882, *p* = 0.0166). Values of *p* in the figure were calculated by Sidak’s multiple comparison test.

In order to examine if trigeminal ganglion neurons play a role in mild hypoxia-induced arousal, we counted the number of cells activated in the region under various levels of oxygen concentrations (Figure 4). For mild hypoxia, we selected 13% O_2_ (Figure 4A) because the previous experiment showed that half (three out of six) of the WT mice woke up at 13% or higher O_2_ but none of the KO mice did (Figure 3E). P-ERK-like immunoreactivity was observed in both neurons (Figure 4B, arrows) and satellite glial cells (Figure 4B, open triangles) in the trigeminal ganglion. To analyze neuronal activation, we counted the number of cells that were positive for NeuN (Figure 4B, closed triangle) and the cells that were positive for both p-ERK and NeuN (Figure 4B, star). Sampling bias seemed minimal because the number of NeuN-positive cells (~1,000/animal) was not different among the groups (effect of genotype, *F*1, 24 = 0.02695, *p* = 0.8710; effect of O_2_ concentration, *F*2, 24 = 0.002347, *p* = 0.9977, two-way ANOVA, *n* = 5 in each group). As expected, exposure to 13 and 10% hypoxia for 3 min in WT mice significantly increased the number of cells that were both p-ERK and NeuN positive (Figure 4C). In KO mice, 10% (*p* = 0.0004, Sidak’s multiple comparison test) but not 13% (*p* = 0.7566) hypoxia activated the trigeminal neurons. Thus, TRPA1 seems to contribute to mild hypoxia-induced activation of trigeminal neurons.

### Genetic Ablation and Pharmacological Inhibition of TRPA1 Reduced Mild Hypoxia-Induced Respiratory Chemoreflex

**Figure 5:**
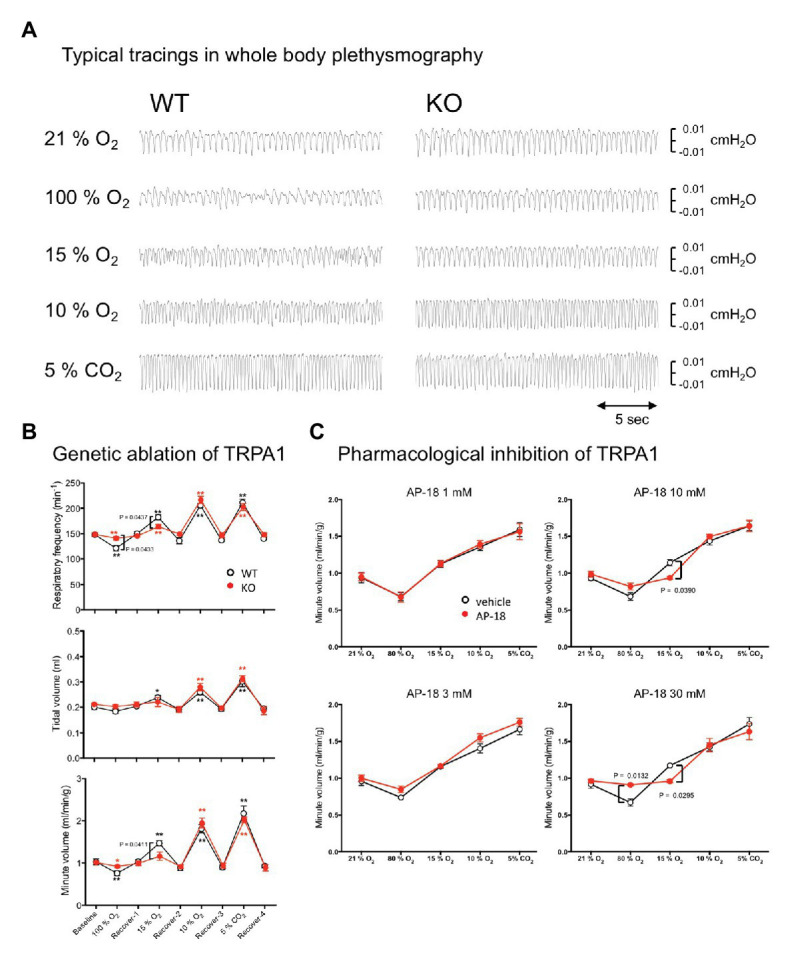
Effect of genetic and pharmacological inhibition of TRPA1 on respiratory chemoreflex. **(A)** Representative tracing of pressure signals in whole body plethysmography. Respiration of WT and KO mice was measured using flow-through type whole body plethysmography. Every gas condition was maintained for 3 min, and the data were collected during the last 20-s period. Each stimulus was separated by intervals of 20 min or more of normal room air. Plethysmographic signal that is a pressure difference between the measuring chamber and the reference chamber, and O_2_ concentration in the measuring chamber were continuously monitored. Data for baseline and recovery periods were obtained during the last 3 min before the next stimulation. **(B)** Group data obtained in whole body plethysmography. Data are shown as mean ± SEM. *n* = 8 for WT and *n* = 8 for KO mice. Two-way ANOVA revealed that there was a significant difference among gas conditions (*F*8, 112 = 101.7, *p* < 0.0001) and interaction between gas condition and genotype (*F*8, 112 = 2.415, *p* = 0.0191). ^*^p < 0.05, ^**^p < 0.01 as compared to preceding control values (one-way ANOVA followed by Sidak’s multiple comparisons test). Values of *p* in the figure were calculated by Sidak’s multiple comparison test to compare between WT and KO mice. **(C)** Using four sets of WT animals (*n* = 5/group), the possible effect of the TRPA1-blocker, AP-18, on respiratory chemoreflex was examined. Every animal was exposed to an aerosol made from vehicle (20% ethanol in saline) and an assigned concentration of AP-18 solution. Data are shown as mean ± SEM. Two-way ANOVA revealed that there was a significant interaction among drugs × gas conditions for 10 mM (*F*4, 16 = 3.668, *p* = 0.0265) and 30 mM (*F*4, 16 = 6.386, *p* = 0.0029) but not for 1 mM (*F*4, 16 = 0.1507, *p* = 0.9600) and 3 mM (*F*4, 16 = 0.7821, *p* = 0.5532). Values of *p* in the figure were calculated by Sidak’s multiple comparison test.

We next examined whether a normal respiratory chemoreflex was intact in TRPA1-KO mice. Hyperoxia (100% O_2_), mild hypoxia (15% O_2_), severe hypoxia (10% O_2_), and hypercapnia (5% CO_2_), separated by intervals with normal room air (21% O_2_), were applied in this order (Figures 5A,B). A similar experimental setting to that shown in Figure 3A was used without EEG and EMG measurement. In WT mice, hyperoxia reduced respiratory frequency and hypoxia and hypercapnia increased it as expected. Tidal volume did not change under hyperoxia but did increase under hypoxia and hypercapnia. As a result, minute volume significantly decreased under hyperoxia and significantly increased under hypoxia and hypercapnia. Baseline values during room air exposure did not differ between KO and WT under any of the respiratory parameters. As for chemoreflex, a qualitatively similar response was observed in KO mice but there was a quantitative difference between KO and WT mice. Namely, respiratory frequency during hyperoxia was significantly higher in KO (141 ± 3 min^−1^) than in WT (121 ± 6 min^−1^, *n* = 8 each, *p* = 0.043, Sidak’s multiple comparison test) and was significantly lower during mild hypoxia (163 ± 4 min^−1^ in KO vs. 183 ± 5 min^−1^ in WT). There was no statistical difference in tidal volume between KO and WT in any condition. As a result, minute ventilation during mild hypoxia was significantly lower in KO (1.15 ± 0.10 ml/min/g) than in WT (1.47 ± 0.06 ml/min/g, *n* = 8 each, *p* = 0.041, Sidak’s multiple comparison test). There was no difference between WT and KO mice with respect to their responses to severe hypoxia and hypercapnia.

We also examined whether abnormalities observed in KO mice can be reproduced by acute pharmacological blockade of TRPA1 in WT mice (Figure 5C). An aerosol of the TRPA1-selective blocker AP-18 was given to mice to attempt an administration that remained localized to the airway. A whole body plethysmographic apparatus similar to the above-mentioned experiment was used with the exception that the gas flow into the recording chamber was stopped for the 3 min of the recording period because the nebulizer responsible for aerosolizing the drug can cause fluctuations in the basal gas flow and result in inaccurate measurements of plethysmographic signals. When compared to the vehicle, 1 and 3 mM of AP-18 did not affect hyperoxic, hypoxic, and hypercapnic responses. When 10 mM of AP-18 was used, the chemoreflex increase in ventilation under mild hypoxia was significantly inhibited (0.94 ± 0.02 ml/min/g for 10 mM AP-18 vs. 1.14 ± 0.04 ml/min/g for vehicle, *n* = 5, *p* = 0.0390, Sidak’s multiple comparisons test). With 30 mM AP-18, hyperoxic depression (0.91 ± 0.01 ml/min/g for 30 mM AP-18 vs. 0.67 ± 0.05 ml/min/g for vehicle, *n* = 5, *p* = 0.0132) and mild hypoxia-induced excitation (0.96 ± 0.02 ml/min/g for 30 mM AP-18 vs. 1.17 ± 0.02 ml/min/g for vehicle, *p* = 0.0295) were significantly attenuated.

Both genetic and pharmacological blockade of TRPA1 indicate its probable contribution to mild hypoxia-induced respiratory excitation and, to a lesser extent, hyperoxia-induced respiratory depression.

### RNA Expression of TRPA1 in the Carotid Body

**Figure 6:**
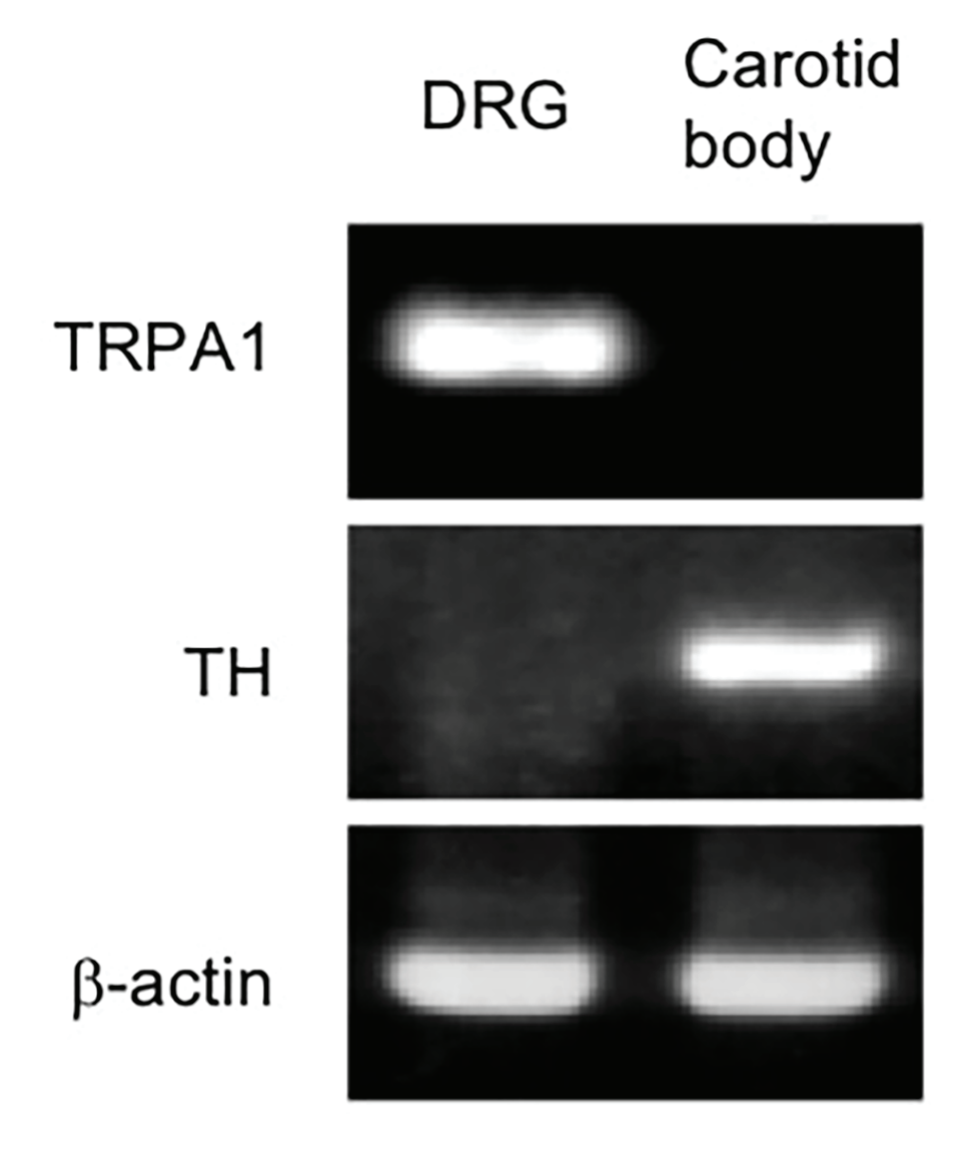
RNA expression of TRPA1 was undetectable in the mouse carotid body. RNAs are subjected to RT-PCR from the mouse carotid body and that from dorsal root ganglia (DRG) as a positive control of TRPA1 expression. Identification of carotid body is confirmed by the detection of tyrosine hydroxylase (TH) RNA, a specific marker for carotid body in the carotid artery region. A similar result was obtained in two mice.

To evaluate possible expression of TRPA1, the carotid body and DRG in WT mice (*n* = 2) were analyzed using RT-PCR (Figure 6). Abundant RNA for TRPA1 was detected in the dorsal root ganglion as expected (Takahashi et al., 2011). Although carotid body expressed RNA for tyrosine hydroxylase, a marker of the hypoxia-sensing glomus cell in the carotid body (Mills et al., 1978), TRPA1 RNA was not detected in our protocol.

## Discussion

We report here that TRPA1-like immunoreactivity in the nasal cavity is in line with trigeminal distribution of TRPA1. In addition, using TRPA1-KO mice, we show three lines of evidence that TRPA1 is indispensable for multiple mild hypoxia-induced physiological responses. First, KO mice showed a significantly attenuated avoidance behavior in response to a low (15%) oxygen environment. Second, the wakeup response to a hypoxic ramp (from 21 to 10% O_2_ in 40 s) was measured using EEG electrodes implanted in the mice. While WT mice awoke at 13–14% O_2_ within 30 s, KO mice did not wake up until O_2_ reached 10%. Histological analysis confirmed a decrease in the amount of trigeminal neuronal activation caused by mild hypoxia in KO mice. Third, KO mice showed attenuated chemoreflex ventilatory responses to mild hypoxia (15% O_2_) but not severe hypoxia (10% O_2_). Similar responses were observed in WT mice treated with an aerosol of the TRPA1 blocker, AP-18 (10–30 mM). These data clearly show that TRPA1 is indispensable for multiple mild hypoxia-induced physiologic responses. We propose that TRPA1 expressed in the sensory nerves along the airway plays a protective role against hypoxia, which presumably occurs before systemic and/or cellular hypoxia takes place.

Although our results indicate a role for TRPA1 in hypoxia-induced physiological responses, it was restricted to mild hypoxia (15% O_2_). Severe hypoxia (10% O_2_) evoked similar responses in TRPA1-KO mice to those in WT mice (Figures 2–5). This may be due to the existence of the canonical sensor for oxygen, the carotid body, in TRPA1-KO mice. Although the carotid body can also be activated by mild hypoxia, the relationship between O_2_ concentration and carotid body activation is not linear but rather hyperbolic (Peng et al., 2010). As a result, sensitivity (activation/change in O_2_) is not as high during mild hypoxia as it is during severe hypoxia. In the case of TRPA1, however, sensitivity to hypoxia was relatively linearly related to the change in O_2_ *in vitro* (Takahashi et al., 2011). Hypoxemia must be occurring before the carotid body is recruited, and because cardiac output of mice is reported to be ~20 ml/min (Kreissl et al., 2006) and blood volume around 3 ml (Riches et al., 1973), there would be a delay of approximately 9 s for blood PO_2_ to reach a new equilibrium with inhaled PO_2_. These factors may explain why only mild hypoxia induced differences between TRPA1-KO and WT mice, especially in the delayed arousal in TRPA1-KO mice (Figure 3).

In support for above interpretation about mild vs. server hypoxia induced responses, we found negligible expression level of mRNA for TRPA1 in the carotid body of the WT mice based on RT-PCR (Figure 6). Thus, function of the carotid body in TRPA1-KO mice is expected to be normal. Our observation is in line with the report showing upregulation of TRPC5 in the carotid body as compared to the superior cervical ganglion neurons, which are practically O_2_-insensitive, using microarray analysis (Gao et al., 2017). Nevertheless, further analyses, such as quantitative PCR and western blotting, would be necessary for precise understanding of TRPA1 expression in the carotid bodies.

In studying trigeminal activation during hypoxia (Figure 4C), we expected a lower activation in TRPA1-KO mice in both mild and severe hypoxia because the carotid body may not be involved in this effect. We do not know the precise explanation for severe hypoxia-induced activation of trigeminal ganglion neurons in TRPA1-KO mice at present. One possibility may be HIF mediated cellular activation in the trigeminal ganglion neurons. We showed that hypoxia induced both trigeminal neuronal activation and arousal from sleep. Although there is no evidence for causative relationship between trigeminal activation and arousal, involvement of TRPA1 in both phenomena is evident from the data of TRPA1-KO mice.

We used a conventional knockout model of TRPA1. This animal is supposed to be deficient in TRPA1 throughout the body. Thus, we cannot distinguish any possible roles of TRPA1 in trigeminal nerve and vagus nerve, which are located along the upper and lower air ways, respectively. In addition, we cannot eliminate the possibility of developmental changes in TRPA1-containing cells in KO mice. Therefore, a time-dependent and/or tissue-dependent knockout model (Zappia et al., 2016) and carotid body denervation (Hemelrijk et al., 2019) may be useful in future studies.

The abnormalities in TRPA1-KO mice’s physiological and behavioral responses to mild hypoxia (13–15% O_2_), along with location of TRPA1 in the trigeminal nerve in the nasal cavity, indicate not only a high sensitivity of TRPA1 to hypoxia *in vivo* but also its participation in a feedforward control of oxygen availability. With TRPA1, a normal animal can behaviorally avoid hypoxic environments and increase ventilation before hypoxemia takes place. As a result of these findings, we propose that TRPA1 plays a role as a frontline alarm for environmental hypoxia.

## Data Availability Statement

All datasets presented in this study are included in the article/supplementary material.

## Ethics Statement

The animal study was reviewed and approved by Experimental Animal Research Committee of Kagoshima University.

## Author Contributions

SC, NT, and TK designed the study. SC, JP, NT, and TK wrote the manuscript. All authors conducted the study, analyzed the data, and approved the final version of the manuscript.

### Conflict of Interest

The authors declare that the research was conducted in the absence of any commercial or financial relationships that could be construed as a potential conflict of interest.
